# Rice Husk Ash/Silicone Rubber-Based Binary Blended Geopolymer Coating Composite: Fire Retardant, Moisture Absorption, Optimize Composition, and Microstructural Analysis

**DOI:** 10.3390/polym13060985

**Published:** 2021-03-23

**Authors:** Mohd Salahuddin Mohd Basri, Tee Hui Yek, Rosnita A. Talib, Intan Syafinaz Mohamed Amin Tawakkal, Siti Hasnah Kamarudin, Norkhairunnisa Mazlan, Nurul Ain Maidin, Mohd Hidayat Ab Rahman

**Affiliations:** 1 Department of Process and Food Engineering, Faculty of Engineering, Universiti Putra Malaysia, UPM, Serdang 43400, Selangor, Malaysia; teehuiyek@gmail.com (T.H.Y.); rosnita@upm.edu.my (R.A.T.); intanamin@upm.edu.my (I.S.M.A.T.); 2 Laboratory of Halal Science Research, Halal Products Research Institute, Universiti Putra Malaysia (UPM), UPM, Serdang 43400, Selangor, Malaysia; 3 Laboratory of Biopolymer and Derivatives, Institute of Tropical Forestry and Forest Products (INTROP), Universiti Putra Malaysia, UPM, Serdang 43400, Selangor, Malaysia; 4 School of Industrial Technology, Faculty of Applied Sciences, Universiti Teknologi MARA (UiTM), Shah Alam 40450, Selangor, Malaysia; sitihasnahkam@uitm.edu.my; 5 Department of Aerospace Engineering, Faculty of Engineering, Universiti Putra Malaysia, UPM, Serdang 43400, Selangor, Malaysia; norkhairunnisa@upm.edu.my; 6 Institute of Advanced Technology (ITMA), Institute of Advanced Technology, Universiti Putra Malaysia, UPM, Serdang 43400, Selangor, Malaysia; 7 Faculty of Mechanical and Manufacturing Engineering Technology, Universiti Teknikal Malaysia Melaka, Hang Tuah Jaya, Durian Tunggal 76100, Melaka, Malaysia; nurulain.maidin@utem.edu.my (N.A.M.); mohdhidayat@utem.edu.my (M.H.A.R.)

**Keywords:** rice husk ash, silicone rubber resin, moisture absorption, fire retardant, statistical, response surface methodology (RSM), geopolymer, coating

## Abstract

Geopolymer coating using rice husk ash (RHA) as the aluminosilicate source has shown excellent fire retardant properties. However, incorporation of rice husk ash into the geopolymer matrix increased water absorption properties of the polymer composite. As such, silicone rubber (SiR) was introduced to improve the moisture absorption and fire retardant properties of the composite. Additionally, the less efficient one-factor-at-a-time (OFAT) approach was conventionally used in past studies on the RHA-based geopolymer composite. In understanding the optimum value and significant effect of factors on the fire retardant and moisture absorption properties of the binary blended geopolymer coating composite, the use of statistical analysis and regression coefficient model (mathematical model) was considered essential. The objectives of this study are to identify the significant effect of factors on moisture absorption and fire retardant properties, to determine the optimum composition, and to study the microstructure of the rice husk ash/silicone rubber (RHA/SiR)-based binary blended geopolymer coating composite. The RHA/AA and SiR/Ge ratios were chosen as factors, and the response surface methodology (RSM) was employed to design experiments and conduct analyses. Fire retardant and moisture absorption tests were conducted. A scanning electron microscope (SEM) was used to observe the microstructure of geopolymer samples. The RHA/alkaline activator (AA) and SiR/Ge ratios were shown to have a significant effect on the responses (temperature at equilibrium and moisture absorption). The high ratio of RHA/AA and SiR/Ge resulted in a lower temperature at equilibrium (TAE) below 200°C and at moisture absorption below 16%. The optimum formulation for the geopolymer coating composite can be achieved when the RHA/AA ratio, SiR/Ge ratio, and sodium hydroxide concentration are set at 0.85, 0.70, and 14 M, respectively. SEM micrographs of samples with good fire retardant properties showed that the char residue of the geopolymer composite coating, which is a layer of excess silicone rubber, is porous and continuous, thus providing a shielding effect for the layer of geopolymer underneath. The sample with good moisture absorption showed the formation of a thin outer layer of silicone rubber without any cracks. The unreacted SiR formed a thin layer beneath the geopolymer composite matrix providing a good moisture barrier.

## 1. Introduction

Rice is mostly produced in Asia and Asians consume more than 80% of the world’s rice. The volume of rice production is estimated to be 156 million tons per year, thus generating a massive amount of solid waste [1]. Rice husk is the outer cover of the rice grain, which is usually discarded as the main solid waste in rice processing. Rice husk ash (RHA) is obtained by burning the rice husks at 600°C for 6 h and grinding it using a jet mill to reduce the particle size [2]. The rice husk ash has a silica content as high as 90%. Rice husk constitutes about 20% of the weight of whole rice with a reduction in weight from 100 kg of whole rice to about 20 kg of rice husk. The rice husk comprises 50% of cellulose, 25–30% of lignin, 15–20% of silicate, and 10–15% moisture content [3]. It is simultaneously an environmentally friendly and low cost source compared to conventional materials for improving mechanical, thermal, and physicochemical properties of polymer composites [4].

Geopolymer is an amorphous solid inorganic polymer with a tri-dimensional aluminosilicate structure produced from the chemical reaction known as geopolymerization between the alkaline solution and aluminosilicate source [5]. Biomass wastes such as rice husk ash and palm oil fuel ash have been recycled as sources [6,7]. Other common sources are metakaolin [8,9], fly ash [10], and ground granulated blast-furnace slag (GBBS) [11]. Geopolymer coating using RHA as the aluminosilicate source has shown excellent fire retardant properties. Mohd Basri, Mustapha, Mazlan, and Ishak [6] studied the fire retardant performance of rice husk ash-based geopolymer coated mild steel. Five factors including the ratio of alkaline activator (AA), ratio of RHA/AA, curing temperature, curing time, and concentration of sodium hydroxide, were analyzed using a statistical analysis to identify the significant factors that most influence the fire retardant performance of RHA-based geopolymer coating. Results showed that the incorporation of RHA provided the best performance on fire resistance, which is augmented with good adhesion and flexural properties.

Abdul Rashid et al. [12] examined the fire resistance performance of composite coating with geopolymer-based bio-fillers for lightweight panel application. The coating formulation was optimized in terms of thickness, alkaline activator ratio, and curing time which produced the RHA-based geopolymer coating that strongly adhered to the substrate for almost 2 h during the flame exposure test. They concluded that the geopolymer-binder type composite is a potential fire-resistant coating for structural insulated panels. Other researches revealed outstanding fire retardant properties of geopolymer when incorporated with rice husk ash [13,14,15].

Although studies on geopolymer have shown excellent fire retardant properties, research on the moisture absorption of geopolymer coating is extremely limited. The study on moisture absorption is extremely important to widen its potential as an external application rather than limited to an interior coating material. In addition, due to the hygroscopic nature of RHA, incorporation of this waste material in geopolymer increased water absorption [16]. Buyondo et al. [17] studied the effect of rice husk ash loading on the water absorption properties of RHA/Metakaolin geopolymer cement. Results showed that the optimum RHA loading of 11.67% resulted in 22.56% of water absorbed. Liang et al. [18] investigated the water absorption rate of alkali-activated metakaolin geopolymer incorporated with rice husk ash at different RHA loadings (0, 10, 20, 30, 40, and 50 wt%). Findings revealed that the rate of water absorbed decreased from 0 to 20 wt% and subsequently increased when more RHA was added. Zhu et al. [19] studied the influence of rice husk ash on the waterproof properties of ultrafine fly ash (UFFA)-based geopolymer. The UFFA was partially replaced with RHA following five different loadings namely 10, 20, 30, 40, and 50 wt%. It was found that the increase in RHA content from 0 to 20% achieved a significant improvement on the waterproofing property of geopolymer. However, excessive RHA addition resulted in an increase in water absorption.

Rice husk was found to be able to absorb water ranging from 5 to 16% of unit weights, and the unit weight of rice husk is 83 to 125 kg/m^3^ [20]. When incorporating rice husk into concrete, the concrete specimens absorbed more water and have an increased air content with the increasing rice husk loading [21]. Hua et al. [22] studied the water absorption properties of rice husk powder/PLA composites and found that the water absorptivity of materials increased with the increasing of rice husk powder content after 24 h. Ismail et al. [23] investigated the effect of increased loading of white rice husk ash (WRHA) in the silica/WRHA weight ratio of polypropylene/silica/rice husk ash hybrid composites. They found that the water absorption of the composites increased with the increasing silica content in the silica/WRHA weight ratio. Marques et al. [24] researched the behaviour of polymer-based composite materials produced with rice husk and expanded cork by-products. In terms of water absorption capacity, higher values are obtained when higher amounts of rice husk are used.

Since the presence of rice husk ash resulted in an increase in water absorption ability, silicone rubber was introduced in the polymer composite to enhance this property. Silicone rubber can be categorized as an organo-silicon compound since it contains both inorganic and organic components. Its extraordinary properties, relative to other organic rubbers, are due to the Si-O silicone bond and its inorganic properties [25]. Silicone rubber composites have excellent antipollution flashover insulating properties. Its high hydrophobicity leads to a low energy surface affecting any pollution layer. Therefore, the water absorbed by this layer surface remains as small water droplets rather than a continuous water film covering the surface [26].

Recent research developments have led to the alteration of silicone rubber (SiR) to improve its resistance to water absorption and hardness. Silicone rubber filled with various mineral fillers such as calcium carbonate, silica, and wollastonite minerals (CaCO_3_, SiO_2_, and CaSiO_3_) was compared for hardness and water absorption. Silicone rubber-based composites showed good water absorption due to their good binding interaction between the silicone rubber and mineral fillers. The SiR/CaSiO_3_ was the most significant in improving water resistance properties [27]. Pei et al. [28] studied the water absorption properties of cyanate ester resins modified by fluoride-containing and silicone-containing components. Results revealed that the moisture absorption value of the cyanate ester resins incorporated with silicone resin exhibited only 0.39% moisture uptake after 45 days, which was remarkably lower than that of neat cyanate ester resins with 0.98%.

Despite the fact that many studies on fire retardant and moisture absorption properties of composites have been conducted, research on the effect of factors on the fire retardant and moisture absorption properties of rice husk ash/silicone rubber-based binary blended geopolymer is rather limited. Studies conducted to identify the impact of several factors on the properties of the binary blended geopolymer are still minimal. Furthermore, the improved method in determining the optimum formulation using statistical optimization has not been applied in previous studies.

Despite a large number of publications on rice husk ash and silicone rubber-based polymer composite reported in the literature in recent years [29,30,31,32,33], the majority of studies were carried out in the one-factor-a-time (OFAT) approach. For greater efficiency, the application of statistical analysis and regression coefficient (mathematical model) are necessary for better prediction on inquiries such as the optimum composition of binary blended geopolymer coating composite with improved fire retardant and water absorption properties. Compared to OFAT, the design of experiment (DOE) has many advantages, including low requirements for resources (experimental runs, time, material, and manpower), accurate measurement of main effects and interactions, and the ability to simultaneously analyze several variables [34]. Furthermore, the RSM, which was originally coined by Box and Wilson [35], is used commonly as a mathematical model for enquiring into significant effects, interactions, and optimization studies. The central composite design (CCD) has been found to be the best model for the analysis [36]. Table 1 shows the difference in the total number of experimental runs between the full design of RSM and full factorial (classical method) design based on five-level factors. The result shows that for analyzing four factors, a full design of RSM requires only a minimum of 31 experimental runs (one replication) as compared to 625 for a full factorial design [29].

Since the RSM approach, specifically the CDD, has been widely used in polymer optimization [37,38,39,40], it was therefore adopted in this study. The main objectives of this paper are: (i) To identify the significant effect of different factors (RHA/AA and SiR/Ge ratios) on the moisture absorption and fire retardant properties of rice husk ash/silicone rubber-based binary blended geopolymer coating composite, (ii) to determine the optimum composition for the blended geopolymer coating composite, and (iii) to study the microstructure of the blended geopolymer coating composite.

## 2. Materials and Methods

### 2.1. Factors and Levels of the Design of Experiment (DOE)

In the study, the ratio of rice husk ash/alkali-activated (RHA/AA) and silicone rubber/geopolymer (SiR/Ge), designated as V1 and V2, respectively, were selected as factors. Based on the preliminary results using a screening process with fractional factorial design (FrFD), other factors such as sodium hydroxide (NaOH) concentration were kept constant at 14 M. Factors and levels used in the DOE are shown in Table 2.

### 2.2. Design of Experiment

At each design stage, five levels and two factors were applied in the CCD and with two replications for a total of 24 experimental runs. The factors were selected based on preliminary lab work, their significant effect on the responses, and working range (workability). Table 3 displays the complete CCD with coded and uncoded levels of these factors. The value for the total block is 1 and the experiments were carried out in a randomized order.

The significance of the main factors and their interactions is calculated using the analysis of variance (ANOVA). The value of 95% was set as the significance level which reflected the *p*-value of 0.05. Based on the value of the correlation coefficient (R^2^), the regression coefficient model (mathematical model) developed in the ANOVA table was used for optimization purposes. To obtain the regression coefficient model, experimental data were fitted with the second-order polynomial model. The general mathematical model obtained from the analysis is shown in Equation (1):

(1) $$ϒ=\beta_{0}+\sum_{t=1}^{3}\beta_{i}X_{i}+\sum_{i}^{3}\beta_{ii}X_{i}^{2}+\sum_{i-1}^{2}\sum_{j=i+1}^{3}\beta_{ij}X_{i}X_{j}$$

where *Y* is the response, *β_0_*, *β_i_*, *β_ii_*, and *β_ij_* are regression coefficients for the intercept, linear, quadratic, and interaction terms, respectively. *X_i_* and *X_j_* are coded values for the independent variables [41].

In generating the optimization plot, several values need to be set. Since the objective was to minimize the TAE, the upper value, which is the maximum acceptable value, was set at 298 °C. The target value, which is the goal to be achieved (the lowest temperature at equilibrium), was set at 206 °C. The maximum value was set as the highest value of TAE, and the target value was close to its lowest value. The setting of the values was conducted using the MINITAB Software. For moisture absorption properties, the highest of the minimum acceptable values were set at 25.7% since the objective was to minimize the moisture absorption of RHA/SiR-based binary blended geopolymer coating composite. The target value for moisture absorption properties was set at 15.4%.

### 2.3. Raw Materials and Sample Preparation

**Figure 1:**
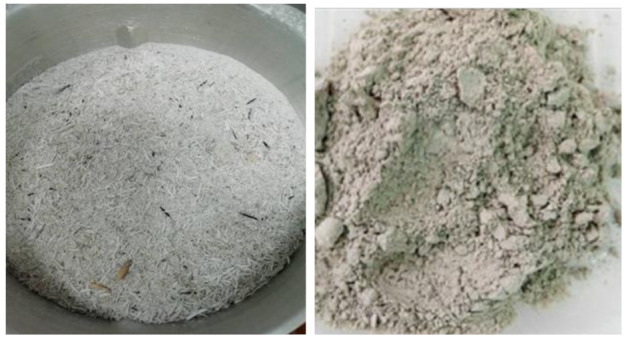
Images of rice husk ash (RHA) before (**left**) and after (**right**) grinding.

RHA was obtained from Maero Tech Sdn. Bhd (Nilai, Malaysia). It was grounded with a blender (MX-SM1031SSL, Panasonic, Malaysia) to finer particle sizes, then sieved (using AS200 Digit, Retsch, Germany) to obtain a particle size with a diameter smaller than 125 microns. Figure 1 shows images of RHA before and after grinding.

**Figure 2:**
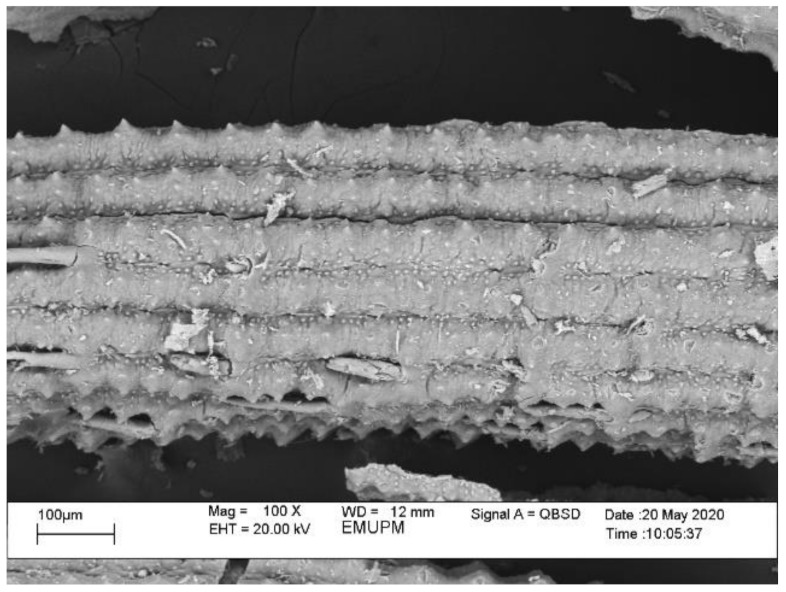
SEM image of the RHA structure before grinding.

The fine structure of RHA before and after grinding viewed under a scanning electron microscope (SEM) is shown in Figure 2. The RHA particle size before grinding ranges from 1 to 100 μm. Particles appear as plates and thin shell-like structures with rectangular indents on the surface. These forms constitute the initial structure of the RH.

**Figure 3:**
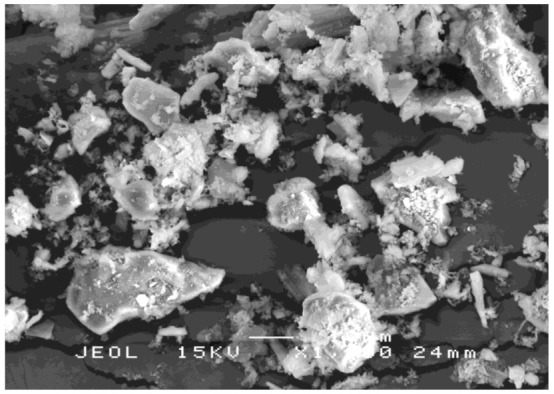
SEM image of the RHA structure after grinding.

RHA has porous, cellular surfaces due to its sponge-like particles. Figure 3 shows the RHA structure after grinding. In a natural solid-state, the particles have a higher concentration of silica with amorphous shapes similar to cristobalite and trace crystalline quartz [42].

The RHA particles must be ground to a very fine particle size to allow for their pozzolanic activity. The condition for burning RH is important in the production of the highest silica RHA in an amorphous state. Conversely, silica derived from unchecked incineration (temperatures higher than 700 °C to 800 °C) mostly comprises cristobalite and tridymite, which are non-reactive silica minerals [43]. The physical properties of the RHA after grinding are given in Table 4.

Sodium hydroxide (NaOH) and sodium silicate (Na_2_SiO_3_) were purchased from Evergreen Engineering and Resources (Semenyih, Malaysia). Silicone rubber was obtained from Malaysia Clay Art (Ampang, Malaysia). The silicone rubber consists of parts A and B, where part A was the catalyzer and part B the crosslinker. Both were mixed with a weight ratio of 1:1 and left aside for curing at room temperature for 3 to 8 h.

Mild steel plates, with a thickness of 1 ± 0.05 mm and dimension of 100 mm in length and 100 mm in width, were cleaned using sandpaper to improve the surface roughness and washed with acetone to remove any unwanted oils or greases. Once the surface was dried at room temperature, the plates were placed in an oven at 45°C for further drying to ensure the excess water is removed. These were then used as a substrate and coated with the rice husk ash/silicone rubber-based binary blended geopolymer coating composite.

**Figure 4:**
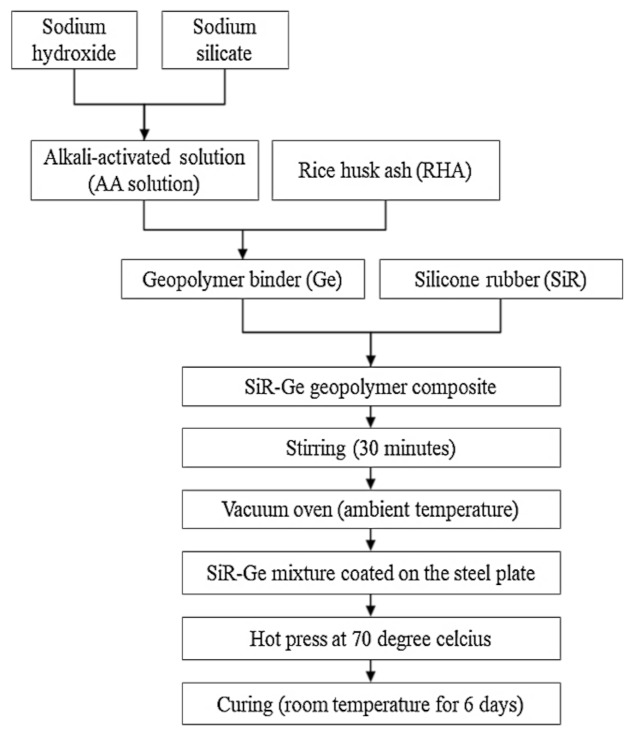
Flow chart for the fabrication of RHA/silicone rubber (SiR)-based binary blended geopolymer coating composite.

**Figure 5:**
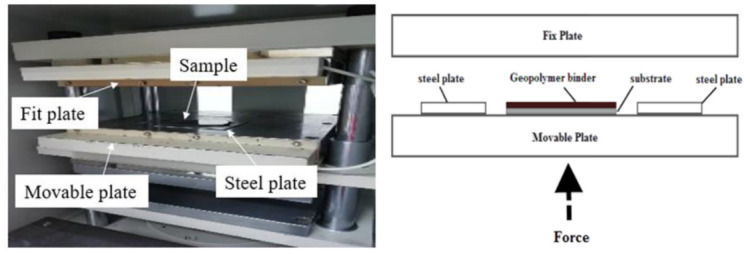
Pre-drying and press process using the hot press machine (**left**) and schematic diagram of the hot press machine (**right**).

Samples were prepared according to the flowchart as shown in Figure 4. The RHA/SiR-based binary blended geopolymer coating composite sample for the fire retardant test was prepared by dissolving a sodium hydroxide (NaOH) pellet in distilled water, followed by the addition of sodium silicate (Na_2_SiO_3_) into the NaOH solution at a ratio of 5.5. The mixture was designated as the AA solution. Subsequently, RHA was added to the solution at a ratio of 0.25, thus forming a dark grey slurry mixture. Silicone rubber was then added into the geopolymer mixture at the ratio of 0.85 to form a binary geopolymer and stirred using a mechanical stirrer (HS-300, WiseStir, Athens, Greece) at 500 rpm for 30 min. The mixture was used to coat evenly over the surface of the mild steel plate. The coated plate was placed in the vacuum oven (Model 53, Binder, Tuttlingen, Germany) to reduce the bubble in the mixture. The plate was then placed into the hot press machine (Gotech, Taichung City, Taiwan) for pre-drying at 70 °C before being pressed to obtain a coating thickness of 1 ± 0.3 mm, as shown in Figure 5. Following this, it is placed in the oven for 24 h at 70 °C for curing. The plate was accordingly left in the open for 6 days at room temperature to complete the curing process.

The dimension for the RHA/SiR-based binary blended geopolymer coating composite sample, for the moisture absorption test, was 3 ± 0.05 mm in thickness and 3 mm in length and width. The preparation process is similar to that for samples in the fire retardant test. During preparation, the binary blended geopolymer mixture was poured onto parchment paper, covered with other parchment papers, and pressed to a 3 mm thickness using a hot press at 70 °C for 3 min. The sample was then cured in the oven at 70 °C for 24 h before being left out for full curing at room temperature over 6 days. The sample was then cut into size.

### 2.4. Fire Retardant Test

**Figure 6:**
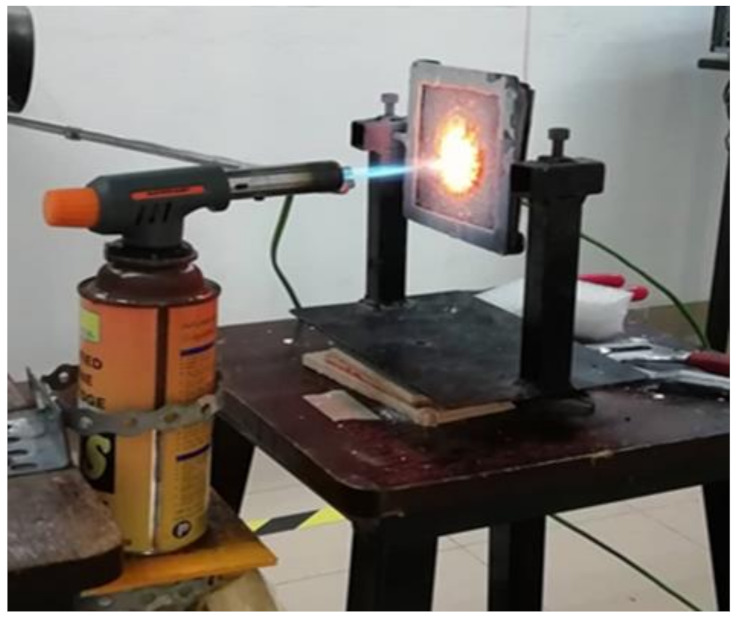
Setup for the fire retardant test.

The test for fire retardant was set up as shown in Figure 6. The test was conducted by heating the coated samples with a direct blow torch flame following the UL-1709 standards [44]. The samples were heated directly using a blow torch with flame temperatures around 900 °C. The flame from the blow torch was fired directly to the center of the sample at a distance of 70 mm. A thermocouple was placed at the back of the sample, where no coating was applied, and connected to a computer that displayed and recorded the surface temperature. A graph of the temperature against time was generated automatically. The test was terminated once the recorded temperature achieves equilibrium.

The ambient temperature and humidity were recorded for each test. The bare mild steel plate was exposed directly to the flame for 10 min for calibration and comparison purposes. As the control sample, coatings without silicone rubber were tested for fire retardant and moisture properties and the results were compared with those of the optimized samples.

### 2.5. Moisture Absorption Test

The moisture absorption test was carried out using the Controlled Humidity Test Chamber according to the ASTM D5229–92 standard [45]. The relative humidity was controlled at 98 ± 1% at room temperature. First, the weight was measured at a stable ambient temperature and recorded as “wet” weight for each sample. The samples were then heated for about 18 h in an oven and the dry weight was measured after cooling down. The process of heating in the oven ensures that the induced moisture absorbed during the fabrication process was removed from the samples. The weight was then described as “dried” weight. During the moisture absorption test, the sample weight was recorded for a maximum of 5 days to reach equilibrium: Initially at every 2 h for the first 24 h, and subsequently followed at 4-h intervals for the next 24 h, then at every 6 h for the next 24 h, and every 24 h for the final 48 h. The percent mass change in each specimen is calculated in accordance to Equation (2), where M% is the percent mass change in the samples, M_t_ is the current mass of the combined fluid and solid at time t, and M_solid_ is the mass of solid measured before the test, at ambient conditions [46].

(2) $$M\%=\frac{M_{t}-M_{solid}}{M_{solid}}\times 100\%$$

### 2.6. Microstructure of Rice Husk Ash

An SEM was used to analyze the difference in the microstructure of samples before and after testing. SEM was conducted using the Hitachi S-3400N variable SEM. A total of nine samples were taken, with three samples before a fire retardant test, three after the test, and another three following a moisture absorption test. The samples were first mounted with a conductive adhesive and sputter-coated with gold-palladium powder. The stub with sample specimens was inserted into the sample chamber of the SEM for viewing. Micrographs of the sample surface were viewed at magnifications of 1000× and 5000×.

## 3. Results and Discussion

The complete design matrix and response values of moisture absorption and TAE are given in Table 5. Data were analyzed using MINITAB. The temperature at equilibrium represents the fire retardant properties of the binary blended geopolymer coating composite. A low TAE indicates that the sample possesses good fire retardant properties and vice versa.

### 3.1. Statistical Analysis of Temperature at Equilibrium and Moisture Absorption Properties

A linear regression model was fitted to the experimental data using the least square technique. Several main parameters were considered in evaluating the statistical results, namely the coefficients of regression, the standard error of coefficient, and the *p*-value of each factor, as well as its interactions (denoted as * symbol) for both responses which are the TAE and moisture absorption. The results in Table 6 indicate that all the factors and interaction effects were highly significant (*p* < 0.000) except for “V_1_* V_2_” with *p* < 0.086. Values for R^2^ = 0.8467 and R^2^ (adjusted) = 0.8041 were considerably high, which indicated that 84.67% of the sample variation in the response was attributed to these factors. The R^2^ value indicates how much of the total variation in the dependent variable is explained by the independent variable. The data perfectly fit the linear model if the R^2^ is 1.0. Any R^2^ value less than 1.0 indicates that the model cannot account for at least some variability in the data [47]. In general, the higher the R^2^, the better the model fits your data. The adjusted R^2^ is a variation of the R^2^ that considers the number of estimated parameters or explanatory variables in a model in relation to the number of data points [48]. Only those independent variables that have an effect on the dependent variable are included in the adjusted R^2^.

For moisture absorption, the *p*-value for both factors (RHA/AA and SiR/Ge ratios) and their interactions were considered significant below the confidence level of 95% (*p* < 0.050). The results shown in Table 7 indicated that all the factors and interaction effects were significant. The *p*-values of all the factors and their interactions were highly significant (*p* < 0.000) except for “V_1_* V_2_” which did not have an effect on the moisture absorption properties of the composite (*p* < 0.201). Values for R^2^ = 0.9459 and R^2^ (adjusted) = 0.9309 were considered very high, which indicated that 94.59% of the sample variation in the response was attributed to the independent variables.

Equations (3) and (4) represent the regression models for the TAE and moisture absorption, respectively.

(3) $${ϒ}_{TAE}=273.146-8.958\left(V_{1}\right)-10.792\left(V_{2}\right)-10.500\left(V_{1}{}^{2}\right)-9.313\left(V_{2}{}^{2}\right)+6.875\left(V_{1}V_{2}\right)$$

(4) $${ϒ}_{MA}=19.031-1.925\left(V_{1}\right)-2.033\left(V_{2}\right)+0.365\left(V_{1}{}^{2}\right)+0.465\left(V_{2}{}^{2}\right)+0.375\left(V_{1}V_{2}\right)$$

where *Y_TAE_* and *Y_MA_* represent the responses which are the temperature at equilibrium and moisture absorption, respectively whereas *V_1_* and *V_2_* are the decoded values of the RHA/AA ratio and SiR/Ge ratio, respectively. The regression models can be used to calculate and analyze the effect of factors on the properties of RHA/SiR-based binary blended geopolymer coating composite.

### 3.2. Effect of Factors on Moisture Absorption and Temperature at Equilibrium

**Figure 7:**
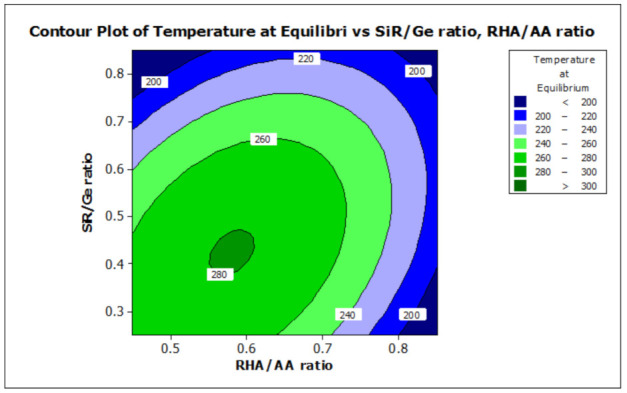
Contour plot for the effect of RHA/AA and SiR/Ge ratios on the temperature at equilibrium properties of RHA/SiR-based binary blended geopolymer coating composite.

**Figure 8:**
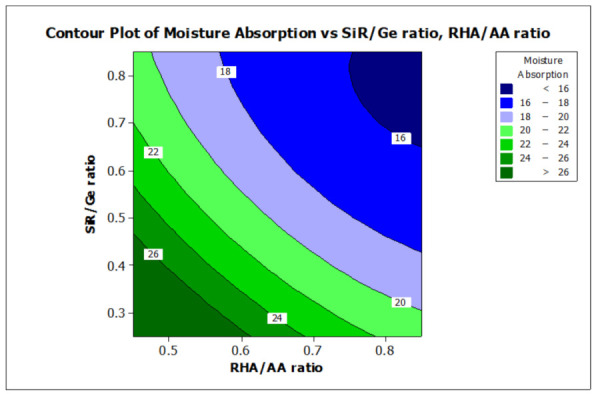
Contour plot for the effect of RHA/AA and SiR/Ge ratios on the moisture absorption properties of RHA/SiR-based binary blended geopolymer coating composite.

ANOVA and regression models were used to analyze the effect of various factors on the properties of the RHA/SiR-based binary blended geopolymer coating composite. Contour plots were used for better illustration. Figure 7 and Figure 8 illustrate the effect of the RHA/AA (V1) and SiR/Ge ratios (V2) on the responses, respectively. A higher V1 and V2 resulted in a lower TAE below 200 °C and lower moisture absorption below 16%. Nasruddin et al. [49] indicated that the bonding structure of the geopolymer became more compact and less porous when a higher RHA/AA ratio of 1.5 was used as compared to that of 1.0. A high ratio of silicon dioxide to aluminium oxide (SiO2/Al2O3) also produced better thermal stability in the RHA geopolymer coating. The effect was due to the high amorphous silica and low aluminium oxide content in RHA after a calcination process [50].

A higher silica content resulted in improved thermal properties of a material [51]. In addition, calcination at a high temperature above 800 °C resulted in a tremendous decrease in the number of black particles, while the number of white creamy silica particles represented by the bright appearance of ash increased. This is due to the fact that as the burning temperature increases during the calcination process, the oxidation of carbon improves. Moreover, the metallic and nonmetallic impurities in RHA including aluminium oxide will be lost more easily at higher temperatures [52]. Additionally, RHA is a porous material and has a high surface area which causes significant absorption of moisture even though it has good fire retardant and mechanical properties. The increased sodium silicate content in the RHA/AA ratio increases the absorption of water due to an increase in the pore size of the geopolymer. Figure 8 shows that the moisture absorption is decreased with an increase in the RHA/AA ratio.

Figure 7 and Figure 8 show that the use of high volume silicone rubber can significantly improve the fire retardant and moisture-resistant properties of geopolymer coatings, which subsequently achieve a lower value of temperature at equilibrium and at moisture absorption. This is due to the silicone rubber content which mainly contains methyl functionality with a few mol percent of vinyl, which can improve high-temperature stability and low-temperature flexibility [53]. According to Khan et al. [54], the incorporation of silicone rubber and nano-SiO^2^ particles may increase the temperature of degradation, resulting in thermal stability. The interactions between nanoparticles and silicone rubber and the increase in physical and chemical crosslinking points are likely to contribute to the improvement of thermal stability [55]. The stability in terms of water absorption is attributed to the good binding interaction between silicone rubber and mineral fillers such as SiO_2_ and calcium silicate (CaSiO_3_) for silicone rubber-based composites [27]. In addition, silicone rubber can improve the resistance to moisture due to its good hydrophobicity, unlike silica, which is hydrophilic.

### 3.3. Optimization of the Responses

**Figure 9:**
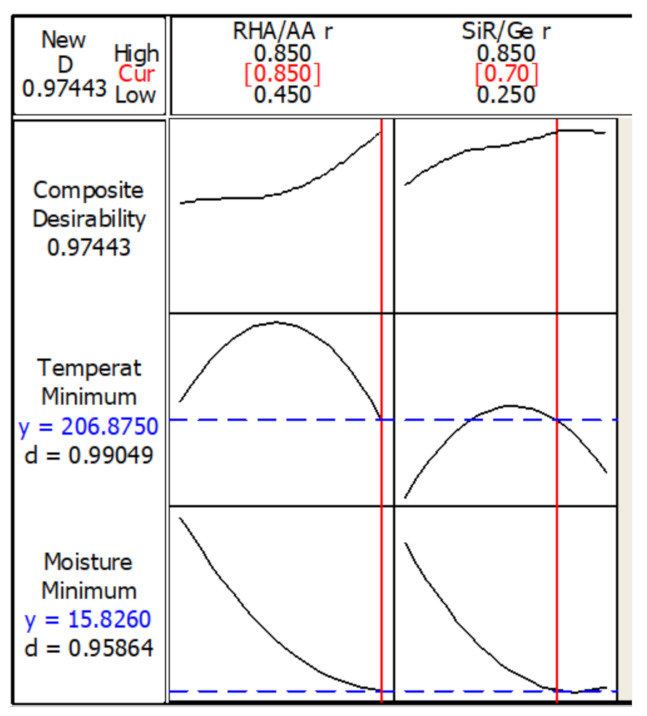
Optimization plot in the temperature at equilibrium and moisture absorption.

Figure 9 shows the optimization plot and the effect of each factor (columns) on the responses or composite desirability (rows). The vertical red lines on the graph represent the optimized factor settings and are displayed by numbers at the top of the column. The horizontal blue lines and numbers represent the responses for the optimized factor level. The optimization was performed under designated parameters.

The optimum TAE and moisture absorption values of 206.9 °C and 15.8%, respectively, can be achieved with the combination of RHA/AA ratio (V_1_) = 0.85 and SiR/Ge ratio (V_2_) = 0.70. The desirability of optimization was calculated as 0.97443, which indicates that all parameters were within the target.

### 3.4. Experimental Validation

From Table 8, it was found that the average error for TAE and moisture absorption was well below 15% at 12.13% and 1.31%, respectively. It was concluded that the developed regression model established using this method was able to optimize the value accurately for the responses.

**Figure 10:**
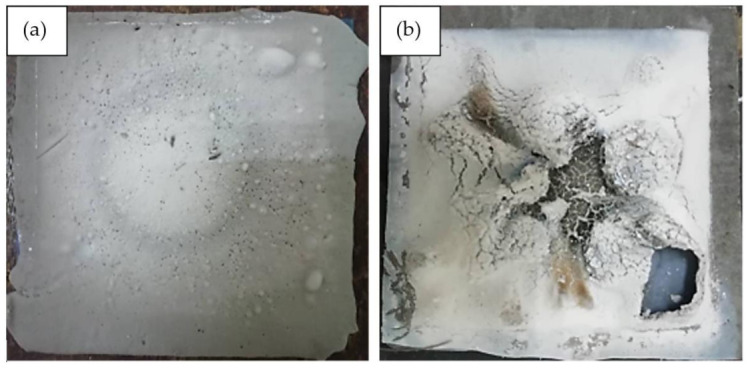
(**a**) Control sample and (**b**) optimized sample after the fire retardant test.

For the control sample, which is without the addition of silicone rubber, the recorded highest temperature at equilibrium and the level of highest moisture absorption was 350 °C and 22.0%, respectively. The results are in agreement with those of other studies which recorded the two values at 350 °C [6] and 30% [56], respectively. The result establishes that the incorporation of silicone rubber improves the fire retardant and moisture absorption properties of geopolymer. Figure 10 shows the surface structure of control and optimized samples following the fire retardant test.

### 3.5. Microstructure Analysis of RHA/SiR-Based Binary Blended Geopolymer Coating Composite

Nine samples were selected to determine the microstructural properties of the RHA/SiR-based binary blended geopolymer coating composite. Three samples were selected after the fire retardant test and compared with three samples of the same mixture before the test, and also three samples before the moisture absorption test. Good, moderate, and poor samples were based on the value of the responses (temperature at equilibrium and moisture absorption) and were compared within the population from the experimental design for RSM. The samples were analyzed using an SEM. The performance of samples based on fire retardant and moisture absorption tests are shown in Table 9.

**Figure 11:**
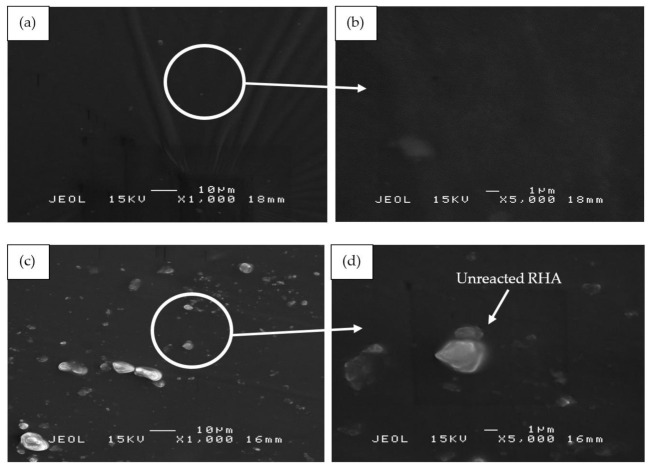

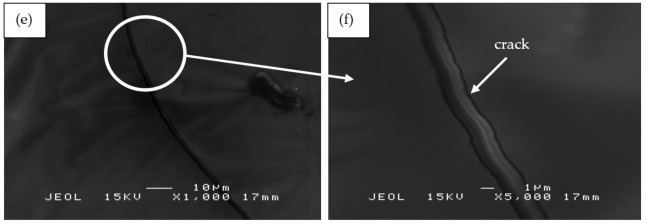
SEM micrographs of RHA/SiR-based binary blended geopolymer coating composite before the fire retardant test with (**a**,**b**) good (sample S17), (**c**,**d**) moderate (sample S18), and (**e**,**f**) poor fire retardant properties (sample S21) at 1000× and 5000× magnifications.

Figure 11 illustrates the SEM micrographs of RHA/SiR-based binary blended geopolymer coating composite before the fire retardant test at 1000× and 5000× magnifications. As shown in Figure 11a,b, sample S17 which possesses good fire retardant properties shows a relatively corrugated wrinkled surface that developed on the surface of the coating. This may probably be due to the improper pressing processes during fabrication and curing. At a higher magnification sample S17, which contained the highest amount of silicone rubber (SiR/Ge ratio of 0.85), a very smooth surface exclusively composed of excess silicone rubber (SiR) is shown, which was not able to mix with the geopolymer matrix following the fire retardant test. Therefore, the SiR formed a thin layer that covered the layer of the composite coating matrix underneath it.

In comparison to sample S17, sample S18 in Figure 11c,d showed RHA particles on its surface. Since S18 contained the highest amount of RHA (RHA/AA ratio of 0.75) and relatively high amount of silicone rubber (SiR/Ge ratio of 0.70), a small amount of unreacted RHA became mixed with the silicone rubber within the geopolymer composite matrix itself, thus resulting in excess RHA particles which appeared on the coating surface.

Figure 11e,f shows sample S21 which contained the lowest content of RHA (RHA/AA ratio of 0.55) and silicone rubber (SiR/Ge ratio of 0.40) as compared to that of sample S17 and S18. The silicone rubber is well incorporated into the geopolymer composite matrix. However, due to the dominant geopolymer composite matrix and high water content (as compared to samples S17 and S18), cracks and corrugated wrinkles were developed during the curing and hot pressing process. The effect of water content on crack formation was proven by Wei et al. [57], who concluded that the high water content in the geopolymer matrix resulted in a sudden shrinkage during the curing process, thus forming cracks. The cracks subsequently provided an opening which allowed heat to travel faster into the layers of coating and thus lower the fire retardant properties of the sample.

**Figure 12:**
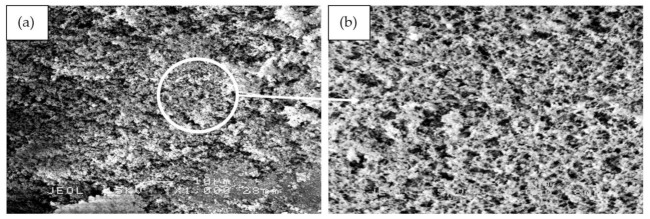

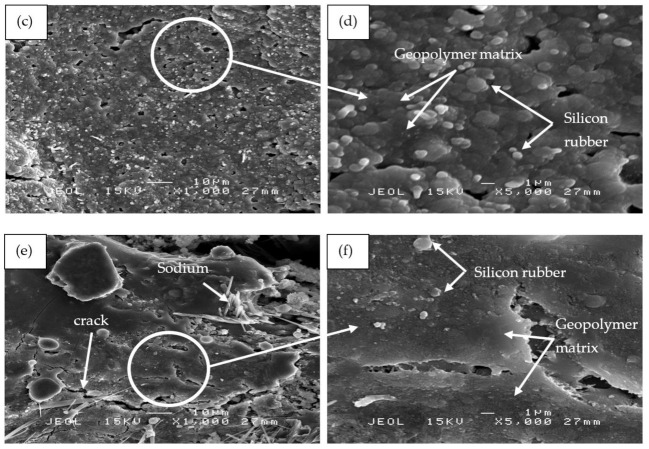
SEM micrographs of RHA/SiR-based binary blended geopolymer coating composite of the residues obtained from fire retardant tests with (**a**,**b**) good (sample S17), (**c**,**d**) moderate (sample S18), and (**e**,**f**) poor fire retardant properties (sample S21) at 1000× and 5000× magnifications.

Figure 12 shows the SEM micrographs of RHA/SiR-based binary blended geopolymer coating composite of the residues obtained from fire retardant tests. As shown in Figure 12a,b, the char residue of the geopolymer composite coating, which is a layer of excess silicone rubber, is porous and continuous. Although there are small holes located in the residue due to permeating gases emerging from the decomposition of silicone rubber, the structure is compact thus providing a shielding effect to the layer of geopolymer composite matrix underneath. The result concurred with that of Zhu et al. [58]. Since the coating has two layers, comprising excess silicone rubber in the top layer and a layer of geopolymer composite matrix beneath, the heat transfer through the small holes was shielded by the geopolymer layer.

The residue char showed in Figure 12c,d is a mixture of silicone rubber and geopolymer composite matrix with a small amount of unreacted RHA. The geopolymer composite matrix is dense with small and large holes scattered in the residue char. The dense structure may be due to the densification of the geopolymer as the binder, and a viscous flow fills most of the voids present in the material [59]. Simultaneously, silicone rubber solidified exothermally by a heat release during the crosslinking reaction [60]. The silicone rubber also provides good adhesion to the cracked surface, producing a bridging effect that keeps the cracked pieces together [61]. The small and large holes allow passage for heat to penetrate and travel directly down to the substrate. According to Li et al. [62], the dense structure may contribute to the formation of a ceramic skeleton and thus considered the coating to be substantially porcelainized.

Figure 12e,f shows sample S21, which exhibited poor fire retardant properties. The surface displays the formation of long and wide cracks in the residual char. Large cracks were initiated from small fissures and later spread out over the surface. As the coating was heated, the water and air inside the coating swelled thus widening the cracks. Excess sodium from sodium hydroxide appeared as long needles scattered on the coating’s surface. Due to the small amount of silicone rubber in the polymer matrix, it was not sufficient to provide additional protection for the substrate.

**Figure 13:**
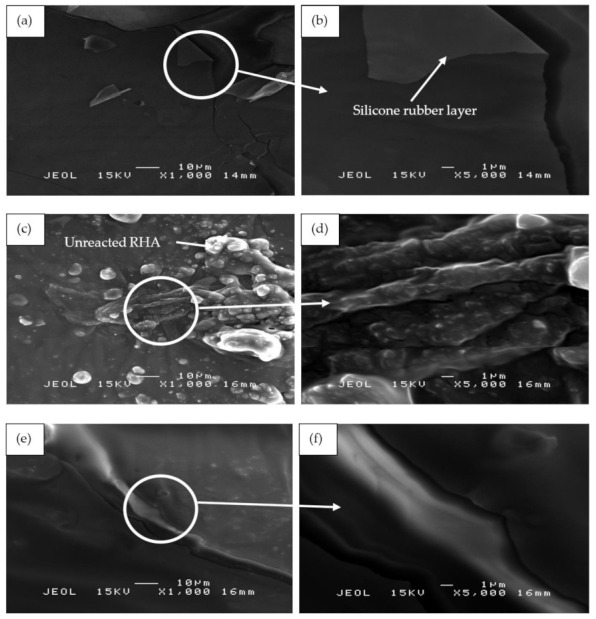
SEM micrographs of RHA/SiR-based binary blended geopolymer coating composite before the moisture absorption test with (**a**,**b**) good (sample S1), (**c**,**d**) moderate (sample S14), and (**e**,**f**) poor moisture absorption properties (sample S21) at 1000× and 5000× magnifications.

Figure 13 shows the SEM micrographs of the binary blended geopolymer coating composite based on RHA/SiR prior to the moisture absorption test. Figure 13a,b reveals that the silicone rubber formed as the outer layer of sample S1 showed no crack formation. A highly viscous geopolymer composite matrix with a RHA/AA ratio of 0.85 molded this layer. Since the content of RHA is higher than those in samples S14 and S21, only a small amount of silicone rubber is able to blend with the geopolymer composite matrix layer leaving the unreacted SiR to form a thin layer beneath it. Since silicone rubber is hydrophobic, it retains only 1% of moisture even after prolonged exposure and thus effectively acts as a barrier against moisture [25].

The unreacted and agglomerated RHA can be seen on the surface of the coating, as shown in Figure 13c,d. The silicone rubber is completely incorporated into the geopolymer composite matrix, with some of it covering the RHA. The unreacted RHA is directly in contact with the moisture and thus increases the rate and percentage of moisture absorption. The agglomerated RHA developed microvoids and gaps between the RHA particles and the geopolymer composite matrix, resulting in a high percentage of moisture absorption.

Figure 13e,f shows the S21 sample, which showed poor moisture absorption properties due to the crack development on the surface of the coating. As reported by Wang et al. [63], cracks and holes formed due to non-uniform mixing during hot pressing consequently increased the moisture absorption rate. When a gap is filled with water, it flows into the adjacent matrix by a capillary action and forms fractured process zones. In these damaged zones, water was absorbed much quicker than in the surrounding undamaged surfaces [64].

## 4. Conclusions

A study on the fire retardant, moisture absorption, optimize composition, and microstructural analysis of rice husk ash/silicone rubber-based binary blended geopolymer coating composite has been successfully performed. The RHA/AA and SiR/Ge ratios were shown to have a significant effect (*p* < 0.050) on the temperature at equilibrium and moisture absorption. Contour plots presented that a high ratio of RHA/AA and SiR/Ge resulted in a lower TAE below 200 °C and moisture absorption below 16%. The optimum value of factors can be achieved when the RHA/AA ratio is 0.85, SiR/Ge ratio is 0.70, and NaOH concentration is 14 M. SEM micrographs of the good fire retardant sample showed that a very smooth thin layer surface of SiR formed, which covered the layer of the composite coating matrix underneath. The sample exhibiting good fire retardant properties showed porous and continuous char residue of the geopolymer composite coating, which provides a shielding effect on the layer of geopolymer composite matrix underlying it. The sample with poor fire retardant properties showed cracks and corrugated wrinkles that developed and which consequently provided openings that allow heat to travel faster into the layers of coating below. SEM micrographs of the sample with good moisture absorption showed that the unreacted SiR formed a thin layer beneath the geopolymer composite matrix, which acts as a barrier against moisture. The sample with poor moisture absorption property formed cracks and holes due to non-uniform mixing and consequently increased the moisture absorption rate.

## Figures and Tables

**Table 1:** Total number of experimental runs for full factorial design and response surface methodology (RSM) based on five-level factors.

Factors	Levels	Total Number of Experimental Runs	
		Full Factorial Design	RSM
4	5	625 (54)	31
5	5	3125 (55)	54
6	5	15,625 (56)	90
7	5	78,125 (57)	160

**Table 2:** Factors and levels.

Factor	Unit	Notation	Levels				
			−2	−1	0	1	2
RHA/AA ratio	-	V_1_	0.45	0.55	0.65	0.75	0.85
SiR/Ge ratio	-	V_2_	0.25	0.40	0.55	0.70	0.85

**Table 3:** Design matrix.

Sample	Coded Factor		Uncoded Factor	
	V_1_	V_2_	V_1_	V_2_
**S1**	2	0	0.85	0.55
**S2**	0	−2	0.65	0.25
**S3**	0	0	0.65	0.55
**S4**	1	−1	0.75	0.40
**S5**	0	0	0.65	0.55
**S6**	0	0	0.65	0.55
**S7**	0	0	0.65	0.55
**S8**	−2	0	0.45	0.55
**S9**	0	0	0.65	0.55
**S10**	0	−2	0.65	0.25
**S11**	0	0	0.65	0.55
**S12**	2	0	0.85	0.55
**S13**	−2	0	0.45	0.55
**S14**	0	0	0.65	0.55
**S15**	1	1	0.75	0.70
**S16**	−1	1	0.55	0.70
**S17**	0	2	0.65	0.85
**S18**	1	1	0.75	0.70
**S19**	−1	1	0.55	0.70
**S20**	0	0	0.65	0.55
**S21**	−1	−1	0.55	0.40
**S22**	1	−1	0.75	0.40
**S23**	0	2	0.65	0.85
**S24**	−1	−1	0.55	0.40

**Table 4:** Physical properties of RHA after grinding.

Properties	RHA
Particles Size	<125 µm
Colour	Light grey
Structure	Power form
Odour	Non

**Table 5:** Design matrix and response value for the temperature at equilibrium and moisture absorption.

Sample	RHA/AA Ratio (V_1_)	SiR/Ge Ratio (V_2_)	RHA/AA Ratio (V_1_)	SiR/Ge Ratio (V_2_)	Moisture Absorption (%)	Temperature at Equilibrium (°C)
S1	2	0	0.85	0.55	16.8	214
S2	0	−2	0.65	0.25	24.6	243
S3	0	0	0.65	0.55	18.4	288
S4	1	−1	0.75	0.40	20.3	252
S5	0	0	0.65	0.55	18.5	283
S6	0	0	0.65	0.55	18.6	280
S7	0	0	0.65	0.55	18.8	270
S8	−2	0	0.45	0.55	24.7	247
S9	0	0	0.65	0.55	18.8	268
S10	0	−2	0.65	0.25	24.3	256
S11	0	0	0.65	0.55	19.1	264
S12	2	0	0.85	0.55	17.3	212
S13	−2	0	0.45	0.55	22.5	244
S14	0	0	0.65	0.55	19.2	260
S15	1	1	0.75	0.70	15.4	230
S16	−1	1	0.55	0.70	19.7	242
S17	0	2	0.65	0.85	17.0	208
S18	1	1	0.75	0.70	16.0	243
S19	−1	1	0.55	0.70	20.2	246
S20	0	0	0.65	0.55	19.5	257
S21	−1	−1	0.55	0.40	25.2	285
S22	1	−1	0.75	0.40	19.1	261
S23	0	2	0.65	0.85	17.0	229
S24	−1	−1	0.55	0.40	25.7	298

**Table 6:** Estimated effects and coefficient for the RHA/alkaline activator (AA) and silicone rubber/geopolymer (SiR/Ge) ratios on the temperature at equilibrium.

Term	Notation	Coefficient	Standard Error of Coefficient	*p*
Constant		273.146	3.460	0.000
RHA/AA ratio	V_1_	−8.958	2.188	0.001
SiR/Ge ratio	V_2_	−10.792	2.188	0.000
RHA/AA ratio* RHA/AA ratio	V_1_* V_1_	−10.500	1.641	0.000
SiR/Ge ratio* SiR/Ge ratio	V_2_* V_2_	−9.313	1.641	0.000
RHA/AA ratio* SiR/Ge ratio	V_1_* V_2_	6.875	3.790	0.086
R^2^ = 0.8467 R^2^ (adj) = 0.8041				

**Table 7:** Estimated effects and coefficient for RHA/AA and SiR/Ge ratios on the moisture absorption.

Term	Notation	Coefficient	Standard Error of Coefficient	*p*
Constant		19.031	0.257	0.000
RHA/AA ratio	V_1_	−1.925	0.163	0.000
SiR/Ge ratio	V_2_	−2.033	0.163	0.000
RHA/AA ratio* RHA/AA ratio	V_1_* V_1_	0.365	0.122	0.008
SiR/Ge ratio* SiR/Ge ratio	V_2_* V_2_	0.465	0.122	0.001
RHA/AA ratio* SiR/Ge ratio	V_1_* V_2_	0.375	0.282	0.201
R^2^ = 0.9459 R^2^ (adj) = 0.9309				

**Table 8:** Experimental validation for the temperature at equilibrium and moisture absorption properties.

Sample	Temperature at Equilibrium (°C)			Moisture Absorption (%)		
	Experimental Value	Predicted Value	Error (%)	Experimental Value	Predicted Value	Error (%)
SV1	248	206.9	19.86	15.73	15.8	0.44
SV2	211	206.9	1.98	15.43	15.8	2.34
SV3	237	206.9	14.55	15.62	15.8	1.14
	$\;\bar{x}$ Error		12.13	$\;\bar{x}$ Error		1.31

**Table 9:** Performance of samples in fire retardant and moisture absorption tests.

**Before and after Fire Retardant Test**			
**Performance**	**Sample Number**	**RHA/AA Ratio**	**SiR/Ge Ratio**
Good	S17	0.65	0.85
Moderate	S18	0.75	0.70
Poor	S21	0.55	0.40
**Before Moisture Absorption Test**			
**Performance**	**Sample Number**	**RHA/AA Ratio**	**SiR/Ge Ratio**
Good	S1	0.85	0.55
Moderate	S14	0.65	0.55
Poor	S21	0.55	0.40

## Data Availability

The data presented in this study are available on request from the corresponding author.
